# Near-Complete Genome Sequence of Pseudomonas palleroniana MAB3, a Beneficial 1-Aminocyclopropane-1-Carboxylate Deaminase-Producing Bacterium Able To Promote the Growth of Mushrooms and Plants

**DOI:** 10.1128/genomeA.00242-18

**Published:** 2018-04-19

**Authors:** Paola Urón, Admir J. Giachini, Bernard R. Glick, Márcio J. Rossi, Francisco X. Nascimento

**Affiliations:** a Departamento de Microbiologia, Universidade Federal de Santa Catarina, Florianópolis, Santa Catarina, Brazil; b Department of Biology, University of Waterloo, Waterloo, Ontario, Canada

## Abstract

The near-complete genome sequence of Pseudomonas palleroniana MAB3, a 1-aminocyclopropane-1-carboxylate deaminase-producing bacterium isolated from an environmental soil Amanita mushroom, is presented here. The genome of P. palleroniana MAB3 contains a single circular chromosome of 6.29 Mb and an average GC content of 60.5%.

## GENOME ANNOUNCEMENT

Ethylene plays an important role in the developmental processes of plants and basidiomycete mushrooms (1). Bacteria producing the enzyme 1-aminocyclopropane-1-carboxylate (ACC) deaminase alleviate the detrimental effects of increased stress ethylene levels in plants (2) and promote the growth and development of ethylene-producing mushrooms by improving their colonization capacity and fruiting structures (3).

In this work, we report the genome sequence of Pseudomonas palleroniana MAB3, an ACC deaminase-producing bacterium isolated from an environmental soil Amanita mushroom (fruit body), which confers significant beneficial effects on both mushroom and plant development.

Genome sequencing of strain MAB3 was conducted following genomic DNA extraction from an overnight culture using a GenElute bacterial genomic DNA kit (Sigma-Aldrich, Germany) according to the manufacturer’s instructions. The DNA library was constructed using the Illumina TruSeq DNA Nano kit (automated) and was sequenced using the MiSeq platform and the MiSeq V3 reagent kit (2 × 300 bp), generating a total of 2,362,402 reads. The initial *de novo* genome assembly was performed using SOAPdenovo version 2.04 (4), which resulted in 16 contigs (all >500 bp). The final 6,291,344-bp genome sequence of strain MAB3 is a scaffold of 16 contigs (*N*_50_ = 505,794 bp), constructed based on a guided assembly against the genome sequence of P. palleroniana BS3265 (= LMG 23076^T^; GenBank accession no. NZ_FNUA00000000) using MAUVE version 2.4.0 progressive alignments (5). The contigs were joined by introducing runs of 100 Ns in the identified assembly gap regions, as indicated in the NCBI submission guidelines. The P. palleroniana MAB3 genome annotation was performed using the NCBI Prokaryotic Genome Annotation Pipeline (6). Functional genome annotation of strain MAB3 was performed using BlastKOALA (7). The annotation of genes involved in secondary metabolite production was conducted using antiSMASH (8).

The genome of P. palleroniana MAB3 contains a single circular chromosome of 6.29 Mb in length with an average GC content of 60.5%. A total of 5,753 open reading frames were predicted, in which 5,679 correspond to protein-coding sequences (CDSs). A total of 74 RNA-related genes were also found.

BlastKOALA analysis resulted in the functional annotation of 3,099 from a total of 5,677 CDSs (54.6%) in which environmental (974) and genetic (647) information processing functions were assigned for most of the annotated CDSs, followed by cellular processes (454) and amino acid (362), carbohydrate (283), cofactor and vitamin (209), energy (203), lipid (112), and nucleotide (103) metabolism. AntiSMASH analysis revealed the presence of several gene clusters involved in the production of nonribosomal peptides, siderophores, bacteriocins, and aryl-polyene. Additionally, genetic traits involved in plant and mushroom growth promotion, such as the ACC deaminase gene and indole-3-acetic acid and cytokinin biosynthesis genes, were found in the genome.

The genome sequence of P. palleroniana MAB3 will bring new insights into the genetics and molecular interactions between beneficial bacteria, mushrooms, and plants, which play a key role in the development of important agricultural and biotechnological applications.

### Accession number(s).

The near-complete genome sequence of P. palleroniana MAB3 has been deposited in GenBank under the accession no. CP025494.
